# 25-Hydroxyvitamin D Depletion Does Not Exacerbate MPTP-Induced Dopamine Neuron Damage in Mice

**DOI:** 10.1371/journal.pone.0039227

**Published:** 2012-07-02

**Authors:** E. Danielle Dean, Lydia M. Mexas, Natalie L. Cápiro, Jeanne E. McKeon, Mahlon R. DeLong, Kurt D. Pennell, Jonathan A. Doorn, Vin Tangpricha, Gary W. Miller, Marian L. Evatt

**Affiliations:** 1 Department of Environmental Health, Rollins School of Public Health, Emory University, Atlanta, Georgia, United States of America; 2 Center for Neurodegenerative Disease, Emory University, Atlanta, Georgia, United States of America; 3 Division of Medicinal and Natural Products Chemistry, College of Pharmacy, The University of Iowa, Iowa City, Iowa, United States of America; 4 Department of Civil and Environmental Engineering, Tufts University, Medford, Massachusetts, United States of America; 5 Department of Pharmacology, Emory University, Atlanta, Georgia, United States of America; 6 Atlanta Veteran’s Administration, Neurology Service, Department of Veterans Affairs Medical Center, Atlanta, Georgia, United States of America; 7 Atlanta Veteran’s Administration, Endocrinology Service, Department of Veterans Affairs Medical Center, Atlanta, Georgia, United States of America; 8 Division of Endocrinology, Diabetes, and Lipids, Department of Medicine, Emory University, Atlanta, Georgia, United States of America; 9 Division of Movement Disorders and Department of Neurology, Emory University, Atlanta, Georgia, United States of America; Virginia Commonwealth University, United States of America

## Abstract

Recent clinical evidence supports a link between 25-hydroxyvitamin D insufficiency (serum 25-hydroxyvitamin D [25(OH)D] levels <30 ng/mL) and Parkinson’s disease. To investigate the effect of 25(OH)D depletion on neuronal susceptibility to toxic insult, we induced a state of 25(OH)D deficiency in mice and then challenged them with the dopaminergic neurotoxin 1-methyl-4-phenyl-1,2,3,6-tetrahydropyridine (MPTP). We found there was no significant difference between control and 25(OH)D-deficient animals in striatal dopamine levels or dopamine transporter and tyrosine hydroxylase expression after lesioning with MPTP. Additionally, we found no difference in tyrosine hydroxylase expression in the substantia nigra pars compacta. Our data suggest that reducing 25(OH)D serum levels in mice has no effect on the vulnerability of nigral dopaminergic neurons *in vivo* in this model system of parkinsonism.

## Introduction

Multiple factors, including increased oxidative stresses, varying genetic susceptibility, mitochondrial dysfunction, proteasomal dysfunction, inflammation and premature apoptosis contribute to the pathophysiology of Parkinson’s disease (PD) [1]. Furthermore, such environmental factors as vitamin D status may modify PD risk and/or progression [2]. Deficient and insufficient vitamin D status has been associated with PD in one case report and several epidemiologic studies [3], [4]. Whether 25(OH)D deficiency states are causal or merely correlative in PD remains poorly understood. Despite this knowledge limitation, many clinicians recommend vitamin D supplementation to patients as a means to prevent or delay progression of PD. Therefore, it is important to clarify the role, if any, that hypovitaminosis plays in PD-related pathology. The goal of this study is to address this gap in our knowledge.

The term “vitamin D” frequently refers to the family of compounds that include the nutritional supplements (cholecalciferol (vitamin D3) and ergocalciferol (vitamin D2)), 25-hydroxyvitamin D (25(OH)D), and the biologically active vitamin D hormone, 1,25-dihydroxyvitamin D (1,25OH_2_D)). Both vitamin D2 and vitamin D3 are rapidly hydroxylated to 25(OH)D, the body’s primary storage form for “vitamin D” and the best indicator of vitamin D status. This 25(OHD) metabolite has much higher circulating concentrations in serum and has a much longer half-life than the serum 1,25OH_2_D, which is considered the hormonal form of vitamin D. The hormonal form of vitamin D, 1,25OH_2_D is dependent on the substrate 25-hydroxyvitamin D. Therefore, circulating 1,25OH_2_D concentrations generally correlate with 25(OH)D concentrations. However, concentrations of the biologically active vitamin D hormone (1,25OH_2_D) are tightly regulated and in our neurology clinical settings it is rare to find patients who have normal renal function and abnormally low 1,25OH_2_D concentrations, even when 25(OH)D concentrations suggest severe vitamin D deficiency.

Several lines of clinical evidence suggest the vitamin D hormone may play a mechanistic role in the pathogenesis of PD. First, genetic factors, such as finding the vitamin D hormone receptor *b* allele BsmI polymorphism is more frequent in patients with PD than healthy controls and 5′ end SNPs on the VDR gene may modulate risk and/or survival in patients with PD [3], [4]. Second, higher 25(OH)D serum levels are associated with reduced risk of developing PD [5]. Third, we and others report that patients with PD are more likely to have 25(OH)D insufficiency (defined as a blood serum level of <30 mg/ml) than aged-matched healthy controls or patients with Alzheimer’s disease [6], [7]. Also, 25(OH)D insufficiency is present early in the disease state (before patients are clinically disabled) [8]. Fourth, in some, but not all cohorts, 25(OH)D levels are lower in patients with more advanced disease, suggesting that as patients’ mobility declines so do 25(OH)D levels or vice versa [6], [9]. These observations have led to speculation that low concentrations of 25(OH)D are involved in PD pathogenesis and/or progression [10].

Laboratory evidence also suggests plausibility that the vitamin D physiology may play a role in the PD. The hormone (1,25OH_2_D) has been shown in multiple injury models relevant to stroke, multiple sclerosis, and Alzheimer’s disease to differentially protect both neuronal populations and glia via upregulation of L-type calcium channels, immune modulation, increased antioxidant function, improved neurotrophin expression [11], [12], [13], [14], [15], [16], [17]. Similarly, 1,25OH_2_D appears to support homeostasis of the dopaminergic neuronal populations affected in PD. Both vitamin D hormone receptors and 1-alpha hydroxylase enzyme (the activating enzyme for vitamin D hormone) are expressed throughout the human brain, but are particularly enriched in the large (likely dopaminergic) neurons of the substantia nigra [18]. These findings have led to speculation that vitamin D deficiency may leave nigrostriatal neurons more vulnerable to insult [10], [19]. Indeed, animal and cell culture studies demonstrate that pre-treatment with vitamin D hormone (1,25OH_2_D) protects dopaminergic neurons from 6-hydroxydopamine and N-methyl-4-phenylpyridinium (MPP+) toxicity [16], [17]. Up to a point, *increasing* vitamin D hormone appears to be neuroprotective. Alternatively, as Newmark and Newmark suggest and as seen in traumatic brain injury models, functional vitamin D deficiency (from reduced 25(OH)D, 1,25OH_2_D or vitamin D receptor dysfunction) may leave nigrostriatal neurons more vulnerable to insult [10], [19], [20]. However, the effect of *reducing* 25(OH)D concentrations (as seen in patients with PD) or vitamin D hormone levels on the vulnerability of the nigrostriatal system to insult has not been tested *in vivo*. To explore this possibility, we determined if 25(OH)D deficiency in mice renders the nigrostriatal system of those mice more vulnerable to insult by the dopaminergic neurotoxin 1-methyl-4-phenyl-1,2,3,6-tetrahydropyridine (MPTP).

## Materials and Methods

### Ethics Statement, Animals and Behavioral Testing

All animal procedures were conducted in accordance with the National Institutes of Health *Guide for Care and Use of Laboratory Animals* and were approved by Emory University Institutional Animal Care and Use Committee for these specific experiments. Retired breeder (8–10 months of age) male C57Bl/6J mice were purchased from Jackson Laboratory. Vesicular monoamine transporter 2 hypomorph (VMAT2 LO) and wild type mice have been previously described [21], [22]. Mice were individually housed on a 12 hour light: 12 hour dark cycle with ultraviolet-free bulbs and were given food and water *ad libitum*. C57Bl/6J mice were randomly assigned to either the control or depleted group. Depleted mice were fed rodent chow devoid of vitamin D (Harlan Teklad Catalog #TD.89123) while control mice were fed a chow containing identical components *plus* vitamin D (Harlan Teklad Catalog #TD.89124). These diets contain 0.47% calcium, 0.3% phosphorus, 16.4% protein, 59.2% carbohydrate, and 10% fat by weight with 3.9 Kcal/g. Therefore, under these conditions, diet (in the control animals) was the only source of vitamin D; depleted animals received no dietary vitamin D and could not make vitamin D from UVB radiation. There was no difference between starting body mass in the control and vitamin D depletion animal groups prior to changing their chow (mean body mass ± SEM- control chow group- 30.44±0.741 grams; depletion chow group- 30.60±0.754 grams). Both groups of mice gained body mass on the new chow formulation and no differences were observed between control-fed and vitamin D depletion chow-fed groups at the time of depletion (mean body mass ± SEM- control chow group- 34.72±1.1 grams; depletion chow group- 33.37±1.1 grams).

### Vitamin D Determination

C57Bl/6J mice were subjected to the control or vitamin D-deficient diet for 6 weeks prior to MPTP challenge. Before MPTP challenge, blood was collected into a BD Microtainer® Serum Separation Tube from the lateral tail vein without restraint. Blood was held on ice for 10 minutes and then centrifuged at 10,000 x g. Serum 25(OH)D levels were assayed by 25(OH)D Direct EIA kit (Immunodiagnostic Systems, Fountain Hills, AZ) per manufacturer’s protocol to confirm depletion. Additionally, tail vein blood was collected from VMAT2 LO and wild type (WT) mice for 25(OH)D measures.

### MPTP Exposure

**Figure 1:**
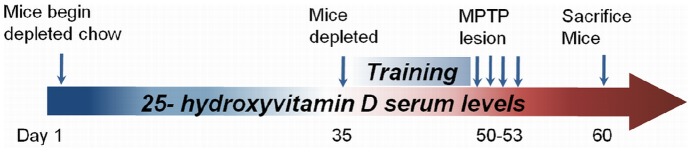
Schematic of experimental design to deplete mice of 25(OH)D levels and challenge with MPTP. On day 1, mice were weighed and randomly assigned to either a group receiving vitamin D depleted chow or a group receiving control chow supplemented with vitamin D. Mice were weighed weekly to check for changes in body mass. After 36 days, 25(OH)D depletion was confirmed by ELISA assay. Then, mice were trained daily to learn the forepaw stride length task from days 44–48. On day 49, baseline behavior was measured. On day 50, MPTP injections began. Mice received a daily injection of either PBS or 15 mg/kg MPTP for 4 days. The mice were allowed to recover for 7 days. On day 60, post-MPTP behavior was measured prior to sacrificing the mice.

A diagram of the study design is shown (Figure 1). For the MPTP lesioning phase, mice received daily subcutaneous injections at the nape of the neck of either phosphate-buffered saline (PBS) or 15 mg/kg of 1-methyl-4-phenyl-1,2,3,6-tetrahydropyridine (as a MPTP-HCl salt Sigma-Aldrich, St. Louis, MO) in PBS for 4 days. Mice were allowed to recover for 7 days after the last MPTP dose and then sacrificed by either live decapitation for tissue collections or by isofluorane anesthetizing and transcardially perfusion with 4% paraformaldehyde in PBS (PFA).

### Motor Behavioral Assessment by Forepaw Stride Length

Forepaw stride length was measured as described previously [23], [24] pre- and post-vitamin D depletion. Individually housed mice were allowed to acclimate to their home cage for a minimum of three days prior to assays. Each mouse was transferred briefly to a novel cage. The home cage was turned on its side at the end of a 2 inch wide by 14 inch long track with white paper lining the bottom. The mice were briefly suspended by the tail over a tray of nontoxic, black ink so the mice could ink their forepaws, were then placed at the beginning of the track opposite their home cage and were allowed to walk to their home cage. Stride length was only measured as a minimum of 4 uninterrupted strides. If the mouse paused during the procedure, the mouse was not allowed to reach its home cage and was put back in the novel cage to repeat the procedure. If a mouse failed to complete the task again, it was excluded from the behavioral assessment. There was no difference between groups for ability to complete the task. Stride lengths were scored as the distance between successive forepaw pad imprints. The average of at least 4 strides was taken for each mouse.

After 25(OH)D depletion was confirmed, mice were trained daily on the task for 5 days. On the 6^th^ day, forepaw stride lengths were collected for pretreatment baseline measures. Mice then received daily subcutaneous injections of either phosphate buffered saline (PBS) or 15 mg/kg of 1-methyl-4-phenyl-1,2,3,6-tetrahydropyridine (MPTP-HCl salt used from Sigma) in PBS for 4 days. Mice were allowed to recover for 7 days. On the 6^th^ day of recovery, mice received an additional day of reinforcement training on the forepaw stride length task. On the 7^th^ day, forepaw stride length measures were taken.

### Western Analyses of Striatal Dopaminergic Markers

Tissues were analyzed by western blotting technique as previously [21], [25]. Unilateral striatum was homogenized with a tissue tearer (Biospec Products, Inc., Bartlesville, OK) in a homogenization buffer of 0.32 M sucrose, 5 mM HEPES pH 7.4 with 1× protease inhibitor cocktail (Sigma Aldrich). Homogenates were centrifuged at 1200×g for 5 minutes at 4°C. The post-nuclear lysate was collected and centrifuged at 20,000×g for 45 minutes at 4°C. The resulting synaptosomal pellet was resuspended in 100 µl of homogenization buffer. Protein concentrations were determined by BCA protein assay (Thermo Scientific). 20 µg of protein lysate was electrophoresed on a NuPAGE 10% Bis-Tris Gel (Invitrogen) and then transferred to a 0.2 µm PVDF membrane (Invitrogen). Blots were blocked in 8% instant milk powder solution (Carnation) for 1 hour at 18–22°C. Primary antibodies (rabbit polyclonal anti-TH and rat polyclonal anti-DAT, Chemicon, Temecula, CA and mouse anti-tubulin, Sigma-Aldrich, St. Louis, MO) were incubated with blots at 4°C for 12–18 hours. Upon several washes, a HRP-conjugated secondary antibody (Sigma) was incubated with the blots for 1 hour at 18–22°C. Blots were washed and then developed with the Super Signal Dura substrate system (Thermo Scientific). Blots were then imaged immediately using a Fluorchem Imaging system (Alpha Innotech). Densitometric analyses were performed using the accompanying software.

### HPLC Analyses of MPP^+^, Dopamine, and Metabolite Levels

An Agilent 1100 Series Capillary HPLC system coupled with an ESA Coulochem III electrochemical detector was used for separation and quantification of dopamine, 3,4-dihydroxyphenylacetic acid (DOPAC) and homovanillic acid (HVA). Briefly, the striatal region of each mouse was weighed and homogenized using a sucrose media (0.32 M sucrose, 10 mM Tris base, 0.5 mM EDTA) at 10% w/v. A 100 µL aliquot was taken and proteins were precipitated by addition of 5 µL perchloric acid. Samples were then spun at 10,000 x *g* for 3 minutes to pellet precipitated proteins. 10 µL of each sample solution was injected and separated using a Thermo Scientific C18 Aquasil column (2.1 x 150 mm, 100 Å). Mobile phase consisted of 50 mM citric acid, 1.8 mM sodium heptane sulfonate, 0.2% trifluoroacetic acid (v/v),pH 3.0 (A) and acetonitrile (B). Gradient conditions were as follows: 3% B at 0 min, 3% B at 8 min, 18% B at 19 min, 3% B at 20 min and 3% B at 35 min using a flow rate of 250 µL/min. Dopamine, DOPAC and HVA were detected using electrodes set at potentials of −150 and +300 mV. Calibration curves were generated using standards to convert peak area to concentration units.

MPP+ levels were measured similarly as described previously [26], [27]. Upon depletion mice were given a single dose of MPTP (20 mg/kg, s.c.) and sacrificed 90 minutes later for striatal dissection as described above. Bilateral striata were used for detection. MPP+ iodide, acetonitrile (≥99.9% HPLC grade), sodium trichloroacetate (TCA, 97.0%) and potassium phosphate monobasic ((≥99.0%) were purchased from Sigma Aldrich (St. Louis, MO). For analysis of MPP^+^ levels, striata were sonicated (Branson S-250A with a double stepped microtip; Danbury, CT) in 5 volumes of 5% TCA and centrifuged for 10 min at 14,000 *g*. MPP^+^ levels were determined by analysis of supernatant using an Agilent Model 1200 HPLC equipped diode array detector operated at a wavelength of 290 nm. Separation was achieved on a reverse phase Altima C18 column (5 um, L = 150 mm, ID = 4.6 mm, Catalog No. 88052; Alltech Associates Inc., Deerfield, IL) with a mobile phase consisting of 89% 50 mM KH_2_PO_4_ and 11% acetonitrile. MPP^+^ was identified by comparison of retention time with known standards and concentrations were calculated from a 6-point standard curve of known concentrations of MPP^+^. Protein concentrations were determined by Bradford Assay.

### Immunohistochemistry of Striatal and Nigral Dopaminergic Markers

Perfused brains were kept at 4°C for 12 hours in 4% PFA. Brains were then transferred to 30% sucrose and stored at 4°C. Forty µm sections were prepared (Microm HM450) and transferred to Tris-buffered saline and stored at 4°C until use. For immunohistochemical staiining, thin sections were washed six times in 1× TBS to remove all traces of PFA. Thin sections were incubated with a rat monoclonal anti-dopamine transporter (DAT) (1∶750; Chemicon), or a rabbit polyclonal anti-tyrosine hydroxylase (TH) antibody (1∶2000; Chemicon) overnight at 4°C and then incubated in a biotinylated-goat anti-rat or goat anti-rabbit secondary antibody (Jackson Immunoresearch, West Grove, PA) for 1 hour at room temperature. Visualization was performed using 0.03% 3,3′-diaminobenzidine (Sigma Aldrich, St Louis, MO) at room temperature.

### Statistical Analyses

All statistical analyses were performed using Graph Pad Prism 5.0. Student t-tests were performed for initial two group comparisons with significant values reported as p<0.05. Two-way AVOVA analyses were performed on all multi-group comparisons with Bonferroni post-hoc tests to determine significant interactions. Significant values are reported as p<0.05.

## Results

### Short Term 25(OH)D Depletion had no Effect the Overall Health or Behavior of the Mice

**Figure 2:**
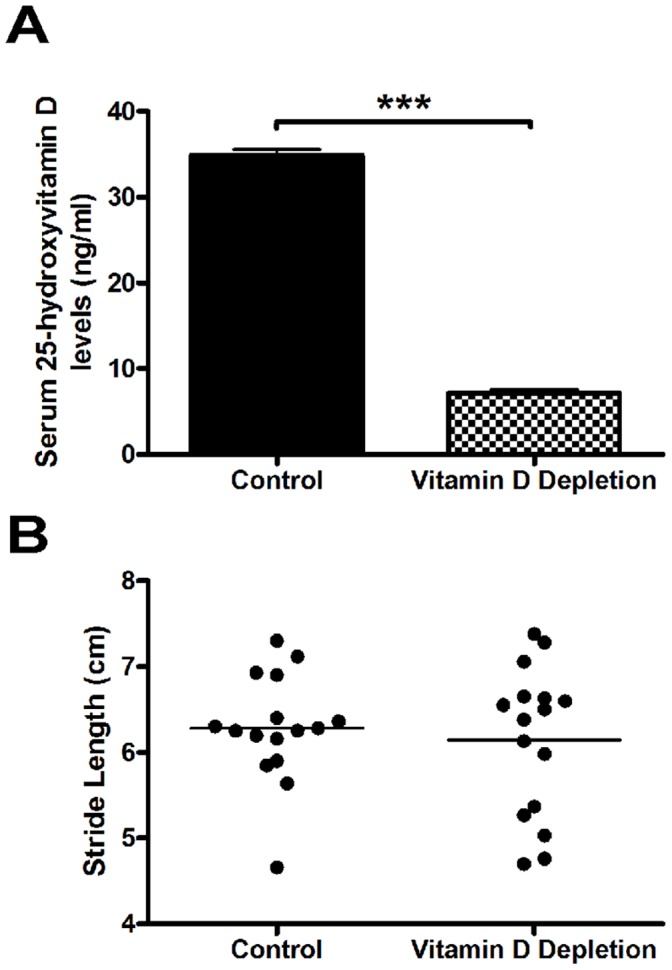
Short term 25(OH)D depletion has no observable effect on mouse locomotor behavior. A) Serum 25(OH)D levels after 6 weeks on either control or vitamin D depletion rodent chow. Results are presented as absolute mean values (ng/ml) ± SEM for eight animals per group (***p<0.0001.) B) Pre-MPTP lesion stride length measures.

In order to test the effect of 25(OH)D deficiency on the vulnerability of nigrostriatal neurons, we first depleted mice of serum levels of 25(OH)D. Since a laboratory animal’s sole source of vitamin D is dietary, mice were transferred from standard rodent chow (Purina) to a vitamin D deficient diet to specifically deplete serum 25(OH)D levels. Serum 25(OH) D levels are widely regarded as the most reliable predictor of vitamin D status due to a longer serum half-life (∼15 days) versus 1,25(OH)_2_D with a much shorter serum half-life (∼15 hours) [28]. Therefore, we used serum 25(OH)D levels to define vitamin D status as it is done in most animal and human studies. After 6 weeks on depleted chow, serum 25(OH)D levels were depleted by nearly 80% (Figure 2A). Vitamin D control chow-fed and depletion chow-fed mice had a mean ± SEM 25(OH)D serum level of 43.9±0.7 ng/ml and 7.17±0.4 ng/ml, respectively (n = 6; p<0.0001).

Next, we confirmed that 25(OH)D deficiency had no effect on the mice’s ability to learn or perform a behavioral index of nigrostriatal damage. Previously, gestational 25(OH)D deficiency has been shown to affect dopamine-dependent locomotor behaviors in mice [29], [30]. However, it is unknown whether 25(OH)D deficiency during adulthood affects motor behaviors. We found that 25(OH)D depletion had no effect on mean forepaw stride length (Control Group- 6.28±0.16 cm; Vitamin D Depletion Group- 6.14±0.22 cm) (Figure 2B). Thus, no overt effect on the mice’s health or behavior during the 6 weeks of 25(OH)D depletion was observed.

### 25(OH)D Depletion did not Further Exacerbate the Effects of MPTP in Mice

**Figure 3:**
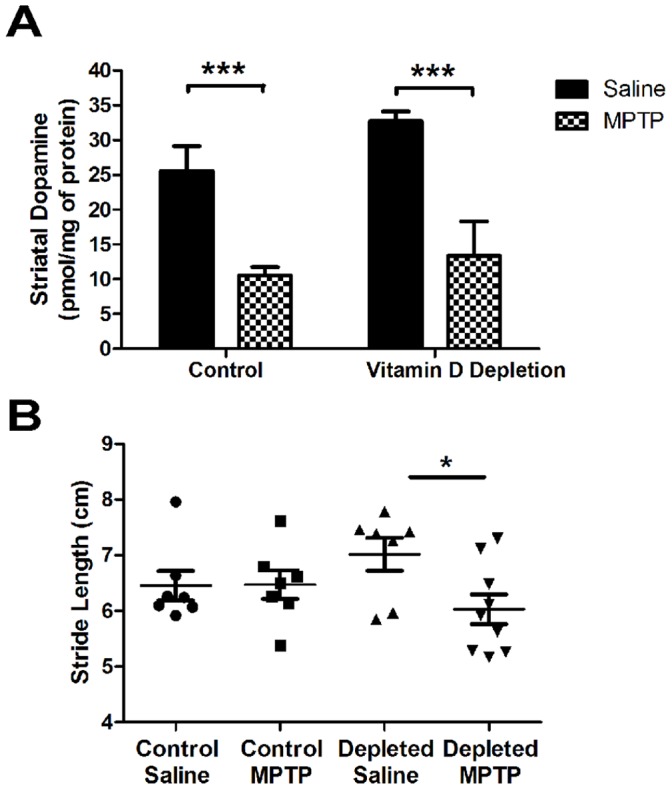
Behavioral and neurochemical effects of 25(OH)D depletion on MPTP susceptibility in mice. A) HPLC analysis of striatal dopamine levels in mice lesioned with MPTP after 25(OH)D depletion. Results are presented as raw values (pmol/mg protein ± SEM; n = 4, ***p<0.001). B) Post-lesion stride length was measured. There are no significant difference control and vitamin D depletion group animals with or without MPTP lesion. The difference within the vitamin D depletion groups is significant (*, p<0.05) with an increase in forepaw stride length in vitamin D depletion group not treated with MPTP over control animals.

We hypothesized that 25(OH)D depletion would not affect the dopamine system alone, rather render the system more vulnerable to future insult. To test this, we challenged 25(OH)D-depleted mice with the dopaminergic neurotoxin MPTP (4 x 15 mg/kg) to produce a mild lesion resulting in approximately 50–60% reduction of striatal dopamine. This dose was chosen so that exacerbation or protection could be detected in the vitamin D-depleted animals. MPTP lesion was confirmed by measuring changes in dopamine and markers of dopamine neuron. Striatal dopamine (Figure 3A) was significantly reduced by 60% in MPTP-lesioned animals as compared to saline-treated animals (Control/Saline- 25.546±3.6 pmol/mg of protein, Control/MPTP- 10.523±1.2 pmol/mg of protein; n = 4). Next, we investigated the effect of 25(OH)D status on the magnitude of dopamine injury. 25(OH)D depletion had no effect on striatal dopamine levels after MPTP lesion. A similar 60% reduction in dopamine levels after MPTP treatment in 25(OH)D depleted animals was observed (Vitamin D Depletion/Saline- 32.735±1.4 pmol/mg of protein, Vitamin D Depletion/MPTP- 13.369±5.0 pmol/mg of protein; n = 4). While 25(OH)D depleted animals trended towards having higher dopamine, there was no significant difference in striatal dopamine levels observed between vitamin D control chow-fed and depleted chow fed animals with or without MPTP lesion.

In order to determine whether 25(OH)D deficiency exacerbates MPTP-induced behavioral changes, forepaw stride length measures were performed. MPTP treatment did not significantly affect forepaw stride length measured at the end of the study (Control/Saline Group- 6.46±0.26 cm; Control/MPTP Group- 6.47±0.26 cm; Vitamin D Depletion Saline Group- 7.02±0.29 cm; Vitamin D Depletion MPTP Group- 5.97±0.25 cm; n = 7−9) (Figure 3B). Interestingly, 25(OH)D deficient-MPTP lesioned mice had significantly shorter forepaw stride lengths than mice that were 25(OH)D deficient-saline treated (*, p<0.05).

**Figure 4:**
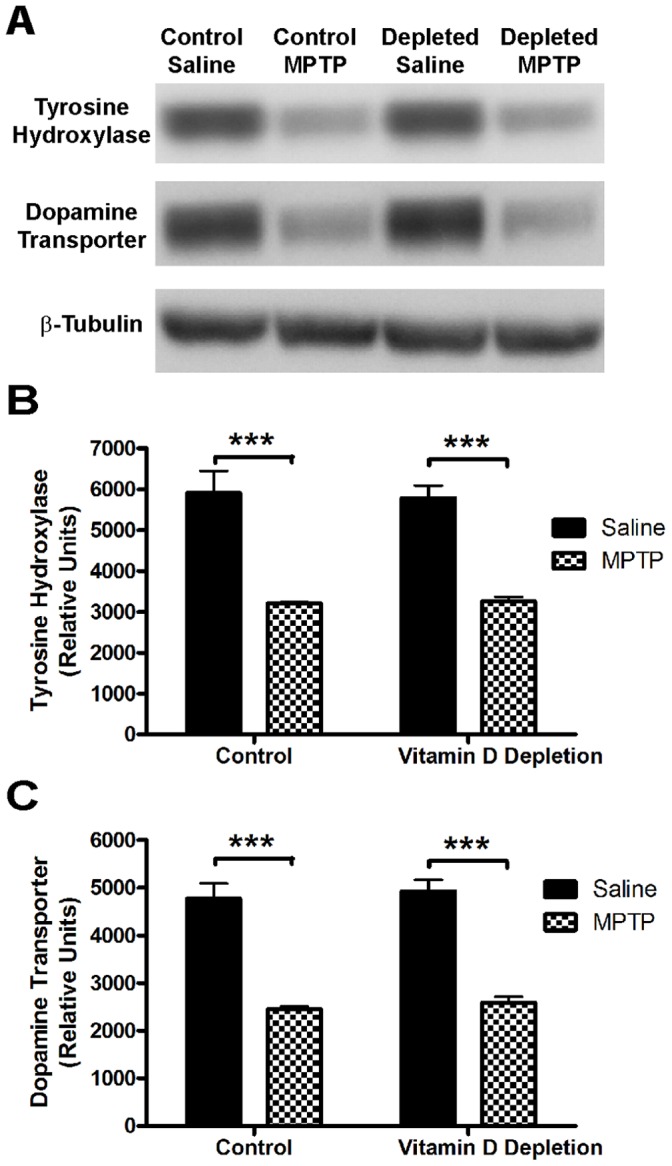
Effects of 25(OH)D depletion on TH and DAT expression in MPTP-lesioned mice. A) Western analyses of striatal TH and DAT levels after MPTP lesion in vitamin D depletion mice. A representative blot is shown. β-tubulin is shown as a loading control. B,C) Densitometric analyses of striatal TH and DAT are shown (Relative values ± SEM; n = 4, ***p<0.001), respectively.

**Figure 5:**
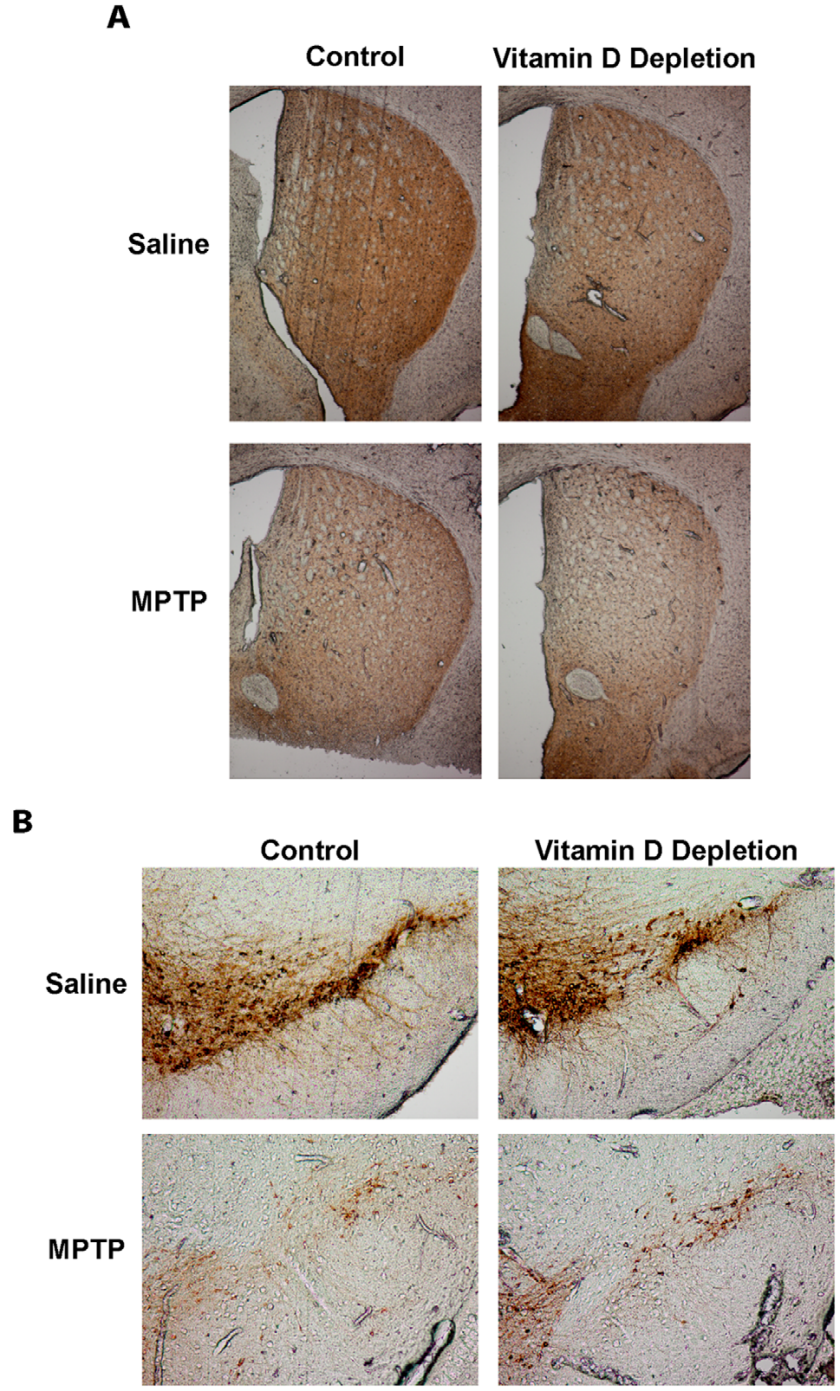
25(OH)D depletion does not exacerbate loss of tyrosine hydroxylase staining in the striatum and nigra after MPTP lesion. A) Representative TH staining of the striatum. B) Representative TH staining of the nigra.

Since changes in markers of dopamine neuron integrity can be seen in the absence of any behavioral deficit and are often changed when dopamine levels are altered, we next investigated the effect of 25(OH)D depletion of expression of markers of dopamine neurons in the nigrostriatal system. A significant 46% and 49% reduction in striatal tyrosine hydroxylase (the rate limiting step in dopamine synthesis) and dopamine transporter protein levels were observed with MPTP lesion, respectively (Figures 4); however, 25(OH)D depletion did not exacerbate the total loss of terminal markers in this paradigm. Additionally, immunohistochemical analyses revealed a dramatic reduction in both of the striatal TH (Figure 5A) and DAT levels (data not shown) and the nigral TH levels (Figure 5B) of MPTP lesioned animals that were not further exacerbated by 25(OH)D depletion. Thus, 25(OH)D deficiency has no effect on expression of markers of dopamine neuron health in mice.

**Figure 6:**
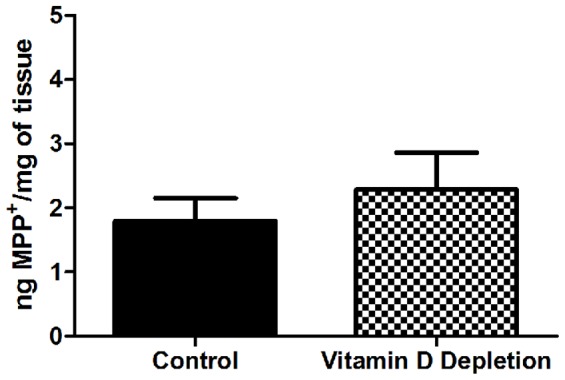
MPP+ levels are not altered by 25(OH)D depletion. Mice were fed vitamin D depleted chow for 50 days and given a single dose of MPTP (20 mg/kg). MPP+ levels were measured by HPLC.

It is possible that 25(OH)D deficiency could affect the metabolism of the MPTP neurotoxin obscuring any effect that 25(OH)D depletion has on the vulnerability of the nigrostriatal system. Therefore, we tested the ability of 25(OH)D deficiency to interfere with MPTP metabolism. In a separate cohort of mice, we found that MPP+ levels were similar in mice challenged with a single dose of MPTP after 25(OH)D depletion (Figure 6). Therefore, MPTP metabolism as measured by MPP+ levels is not altered by 25(OH)D depletion.

### 25(OH)D Levels are not different in Mice with Acute or Chronic Dopamine Deficiency

**Figure 7:**
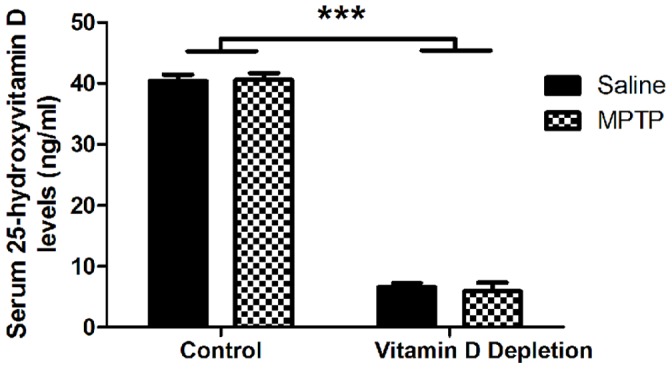
MPTP lesioning does not affect serum 25(OH)D levels. After MPTP lesion, serum 25(OH)D levels were measured to determine if MPTP lesion had any effect (Control/Saline Group- 40.40±1.1 ng/ml; Control/MPTP Group- 40.60±1.1 ng/ml; Vitamin D Depletion/Saline Group- 6.60±0.7 ng/ml; Vitamin D Depletion/MPTP Group- 5.91±1.4 ng/ml; n = 4 (***, p<0.0001).

Because 25(OH)D levels are reportedly lower in patients with more advanced PD, we hypothesized that perhaps acute or chronic dopamine loss drives the lower levels of serum 25(OH)D observed in patients with PD. To test this possibility, we also determined whether dopaminergic neuron damage acutely affected 25(OH)D serum levels. In mice on the vitamin D complete chow, there were no differences in 25(OH)D serum levels between mice treated with MPTP and those treated with saline after ten days of lesion (Control/Saline Group- 40.40±1.1 ng/ml; Control/MPTP Group- 40.60±1.1 ng/ml; Vitamin D Depletion/Saline Group- 6.60±0.7 ng/ml; Vitamin D Depletion/MPTP Group- 5.91±1.4 ng/ml; n = 4; ***, p<0.0001)(Figure 7). However, the half-life of 25(OH)D serum levels are sufficiently long that 25(OH)D concentration changes could have been obscured over the course of only 10 days.

**Figure 8:**
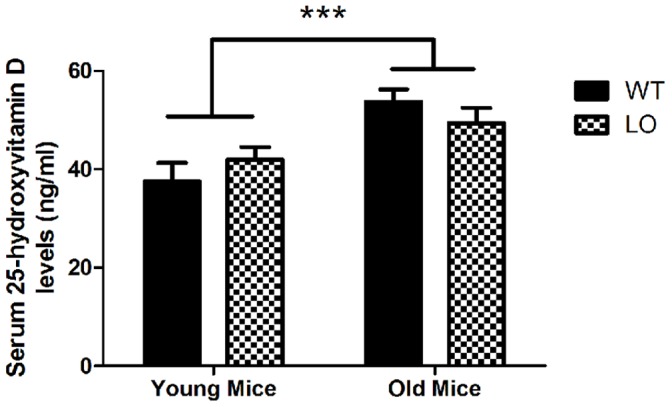
Serum 25(OH)D levels are not changed in VMAT2 LO mice. Serum 25(OH)D levels were measured in both young (2–3 month old) and old (12–15 month) VMAT2 WT and LO mice to determine loss of dopamine has any effect on 25(OH)D serum levels No differences were observed between WT and LO mice; however, older mice have higher serum 25-hydroxyvitamin D levels than young mice (***, p<0.0001).

Therefore to test the effect of chronic dopamine deficiency as seen in PD on 25(OH)D serum levels, we used a mouse model that more closely matches the chronic state of dopamine deficiency, the vesicular monoamine transporter 2 hypomorph (VMAT2 LO) mouse model. These mice have severe impairment of their ability to package dopamine into synaptic vesicles for later release resulting in enhanced turnover of dopamine with a chronic state of hypodopminerigia similar to that seen in patients with PD [21], [22]. We measured serum 25(OH)D levels in both young mice and old mice maintained on standard vitamin D complete rodent chow to detect any changes in 25(OH)D status in these mice. Interestingly, lifelong deficiency in dopamine levels has no effect on 25(OH)D levels (Figure 8). There were no differences in serum 25(OH)D levels in VMAT2 WT and LO mice in young (<3 months) or old (>12 months) mice. Also, older mice had significantly higher serum 25(OH)D levels than younger mice (***, p<0.0001). Therefore, in this model chronic dopamine deficiency does not promote 25(OH)D deficiency when fed a standard rodent chow diet.

## Discussion

With exception of bone and tooth health, the role that vitamin D supplements, 25OHD and vitamin D hormone play in the pathogenesis of disease has been understudied and underappreciated. Because epidemiological data have suggested a link between low 25(OH)D status and PD, we sought to investigate the effects of 25(OH)D depletion in the MPTP mouse model- of parkinsonism. We found that while we are able to effectively reduce serum 25(OH)D levels and lesion animals with MPTP, there was no relationship between serum 25(OH)D levels and the magnitude of MPTP injury as measured by striatal and nigral dopamine neuronal markers, striatal dopamine, and gait analysis. Also, we found no difference in the status of dopaminergic nigrostriatal neurons as measured above with serum 25(OH)D deficiency alone. This suggests that in animals, low serum 25(OH)D levels may not predict dopamine neuronal health. It is unlikely that 25(OH)D deficiency in PD patients is a surrogate marker for some other metabolic changes such as in calcium or parathyroid hormone levels as these levels are rarely altered in our clinical population with 25(OH)D insufficiency (data not shown). It is also unlikely based on previous studies that calcium levels are affected in our model system [31]. While similarly designed studies largely reduce serum vitamin D hormone levels (∼60% reduction), it is possible that vitamin D hormone levels remained high enough throughout our study in 25(OH)D depleted mice to prevent any exacerbation of MPTP toxicity [32]. Additionally, while 25(OH)D concentrations are low early in the course of the disease [8], and in one epidemiologic study lower concentrations of 25OHD were associated with increased risk of PD [5], the duration of 25(OH)D insufficiency in patients with PD prior to diagnosis is unknown. Therefore, to better model chronic 25(OH)D insufficiency (and obtain chronically low-normal or even deficient vitamin D hormone levels), future studies should include longer duration of 25(OH)D depletion in animals.

MPTP is a reliable toxicant used to selectively lesion dopamine neurons and is the only toxicant model available for animal studies that also undoubtedly causes a parkinsonian syndrome in humans [33]. While the MPTP mouse is the most common animal model of PD, it is limited by several factors including peripheral and central metabolism of MPTP to MPP+, expression of DAT on the surface of target cells, and the inability to reproduce the non-motor and progressive symptoms of the disease. We did not detect any effect of 25(OH)D depletion on MPTP metabolism or DAT levels under our experimental conditions (Figures 4 and 6). The MPTP dosing paradigm described in the present study is designed to cause a moderate loss of dopamine and terminal markers, which allows for detection of exacerbation or attenuation of toxicity. Therefore, any effect of 25(OH)D alone on MPTP susceptibility should be revealed, and we feel that we selected the appropriate dosage of MPTP for this study. Additional experiments using a milder 4 x 10 mg/kg MPTP dosing paradigm also revealed no effects of 25(OH)D depletion on MPTP toxicity (data not shown). Chronic Perhaps other models of Parkinson’s disease (such as a transgenic A53T α-synuclein mouse may yield different results [34]. However, chronic dopamine depletion as seen in VMAT2 LO mice did not affect serum 25(OH)D levels (Figure 8) [21], suggesting that dopamine status does not influence vitamin D status.

Previously, we have demonstrated that changes in environmental factors during brain development can predispose the nigrostriatal system to damage later in life [26], [27]. Similarly, during early development, 25(OH)D deficiency profoundly affects brain volume, cell proliferation, and cortical thickness in rats [35]. Additionally, drug-induced hyperlocomotion, increased striatal DAT, and decreased dopamine turnover are seen in rats developmentally deficient in 25(OH)D [29], [30]. This suggests that early deficiency during developmental periods can cause permanent changes to the dopaminergic pathways that may persist into adulthood [36] and future generations [37]. While others have observed changes in dopamine homeostasis in other models of 25(OH)D depletion [23], we observed a small non-statistically significant trend towards increased striatal dopamine in 25(OH)D depleted animals (Figure 3A). When challenged with MPTP, these animals did have a significantly smaller forepaw stride length whereas control chow fed animals had no change (Figure 3B). It is unclear whether the observed differences in dopamine levels and forepaw stride length are a result of 25(OH)D status; however, changes in dopamine homeostasis are strongly correlated to changes in performance in several behavioral tasks, including gait analyses by forepaw stride length [23].

In humans, midlife 25(OH)D insufficiency is associated with higher PD risk while higher serum levels are associated with reduced risk of PD [5]. Whether 25(OH)D insufficiency is causal or correlative remains unknown, but it has been proposed that the vitamin D hormone (1,25OH2D) plays a protective role in the health of the neurons affected in PD and that lower serum levels of vitamin D predispose these neurons to damage. Indeed, pretreatment of rat mesencephalic cultures with 1,25OH_2_D (the biologically active form of vitamin D) confers resistance of dopaminergic neurons to MPP^+^ (the active form of the toxin) [17]. More recently, it was shown that administration of 1,25OH_2_D partially restored TH expression in the nigra of 6-hydroxydopamine lesioned rats [38]. Altering concentrations of 25(OH)D and/or 1,25OH_2_D could be protective by several distinct mechanisms, including stimulating the action of growth factors, acting as an antioxidant, or increasing glutathione production [17], [35], [39], [40]. Interestingly, we did not find the inverse, i.e., that short term 25(OH)D depletion (for eight weeks) made dopaminergic nigral neurons more susceptible to insult by MPTP. Therefore, our data do not support the hypothesis that 25(OH)D status influences dopaminergic neuronal health.

Perhaps studies of other alteration of vitamin D status and models of PD (such as the transgenic A53T α-synuclein mouse or the VMAT2 LO mouse) [21], [34] would yield different results. It is possible that more sustained 25(OH)D deficiency would have led to increased vulnerability to insult, and this paradigm does not lead to changes in other factors associated with chronic deficiency that may be necessary to affect neuronal vulnerability. Alternatively, some interaction between vitamin D status and other genetic factors may affect risk of PD. It is also possible that vitamin D supplementation may protect neuronal populations against insult, but reduced 25(OH)D levels have little or no effect on the viability and vulnerability of dopamine neurons. Thus, additional studies are warranted to explore the role of 25(OH)D deficiency on the long term health of dopaminergic neurons.

### Conclusions

Our data suggest that short term 25(OH)D depletion over the course of a few weeks does not render dopamine neurons more susceptible to the neurotoxin, MPTP as we initially hypothesized. However, additional studies are warranted to explore whether more long-standing deficiency of 25(OH)D or gestational depletion have a role in the laboratory *in vivo* and *in vitro* models of dopaminergic neuronal vulnerability to toxins. These large future studies are necessary to establish the role, if any, that vitamin D supplements, vitamin D status or the active 1,25OH_2_D (hormone) play PD pathophysiology.
